# Debunking the claim that abstinence is usually healthier for smokers than switching to a low-risk alternative, and other observations about anti-tobacco-harm-reduction arguments

**DOI:** 10.1186/1477-7517-6-29

**Published:** 2009-11-03

**Authors:** Carl V Phillips

**Affiliations:** 1 University of Alberta, School of Public Health, 8215 112 St Suite 215, Edmonton, AB, T6G 2L9, Canada

## Abstract

Nicotine is so desirable to many people that when they are given only the options of consuming nicotine by smoking, with its high health costs, and not consuming nicotine at all, many opt for the former. Few smokers realize that there is a third choice: non-combustion nicotine sources, such as smokeless tobacco, electronic cigarettes, or pharmaceutical nicotine, which eliminate almost all the risk while still allowing consumption of nicotine. Widespread dissemination of misleading health claims is used to prevent smokers from learning about this lifesaving option, and to discourage opinion leaders from telling smokers the truth. One common misleading claim is a risk-risk comparison that has not before been quantified: A smoker who would have eventually quit nicotine entirely, but learns the truth about low-risk alternatives, might switch to an alternative instead of quitting entirely, and thus might suffer a net increase in health risk. While this has mathematical face validity, a simple calculation of the tradeoff -- switching to lifelong low-risk nicotine use versus continuing to smoke until quitting -- shows that such net health costs are extremely unlikely and of trivial maximum magnitude. In particular, for the average smoker, smoking for just one more month before quitting causes greater health risk than switching to a low-risk nicotine source and never quitting it. Thus, discouraging a smoker, even one who would have quit entirely, from switching to a low-risk alternative is almost certainly more likely to kill him than it is to save him. Similarly, a strategy of waiting for better anti-smoking tools to be developed, rather than encouraging immediate tobacco harm reduction using current options, kills more smokers every month than it could possibly ever save.

## Introduction

Tobacco harm reduction (THR), the substitution of low-risk nicotine products for cigarette smoking, is increasingly recognized as offering huge public health benefits. Smoking is well known to be a very hazardous activity, but the main reason why people smoke - nicotine - does not itself cause much risk when separated from inhaling smoke. Extensive epidemiology shows that the use of Western oral smokeless tobacco (ST) causes a trivial fraction of the mortality risk from smoking, and it is believed that electronic cigarettes and pharmaceutical nicotine products (gums, patches, lozenges) have similarly low risks. Many smokers will keep smoking until they die from it because, when given only the options of smoking or completely giving up nicotine, many will not give it up. But many of them probably could be persuaded to switch to a low-risk source of nicotine, and the health benefits would be almost as good as quitting entirely.

Readers interested in background on THR that is beyond the present scope, including quantifications of its potential benefits and reports of past successes, can find them in our website [1], in various overview papers (Phillips CV, Heavner K, Bergen P. Tobacco - the greatest untapped potential for harm reduction. Submitted, Available at: ) [2,3], and in endorsements by British and American medical organizations [4,5]. Other relevant contributions to the issue include studies that allow estimates of the potential benefits (Geertsema K, Phillips CV, Heavner K. University Student Smokers' Perceptions of Risks and Barriers to Harm Reduction, Submitted, Available at: ) [6,7], estimates of how much THR has already been employed in the past in the U.S. [8], and how it has largely succeeded in Sweden, where ST has substantially replaced smoking, resulting in the lowest tobacco-related disease rates in the Western world [9,10].

Stated estimates for how much less risky ST is compared to smoking vary somewhat, but the actual calculations put the reduction in the range of 99% (give or take 1%), putting the risk down in the range of everyday exposures (such as eating french fries or recreational driving), that provoke limited public health concern [6]. Even this low risk is premised on the unproven assumption that nicotine causes small but measurable cardiovascular disease risk (as do most mild stimulants such as decongestant medicines, energy drinks, and coffee), since such risks account for almost all of the remaining 1%. Perhaps just as important, even a worst-case scenario puts the risk reduction at about 95%, meaning that any scientifically plausible estimate shows THR has huge potential health benefits. There is no epidemiology for the new electronic cigarettes and very little useful epidemiology for assessing long term use of pharmaceutical nicotine products. But since most of the apparent risk from ST comes from nicotine, and the other ingredients in the non-tobacco products are believed to be quite benign, we can conclude that the risks across these product categories are functionally identical from the perspective of THR.

Because it is not necessary to distinguish among product categories for purposes of the present analysis, a collective description, *THR products*, is used. Product preferences vary and many smokers become attached to aspects of the smoking experience, including the aesthetics (flavor, smell, mouth and airway feel) and social behaviors for which no other product is a perfect substitute. The variety of THR products increases the chance that a given smoker will find one of them a sufficiently good substitute for smoking.

Harm reduction is a generally accepted public health principle that recognizes that eliminating an exposure is often not practical, welfare maximizing, or ethical, and so we should endeavor to reduce the harm from the exposure. The best example is encouraging the use of seatbelts without trying to curtail exposure to automotive transport. However, for politically controversial exposures (e.g., injection drug use, sexual activity outside of marriage, tobacco use) opponents of harm reduction often try to defend their beliefs that "just say no" (abstinence only) is the only acceptable option by observing that "lower risk does not mean no risk". But in the absence of quantification, this observation is merely a trivial vocabulary lesson, not a useful contribution to decision making. The present analysis offers a quantification that illustrates how a 99% reduction in risk is so close to zero risk that the "let's wait and see if we can do even better than current low-risk options" attitude is clearly killing more people than it could ever save. Rational decision strategies call for taking advantage of existing knowledge at some point, rather than continuing to search. If a risk is low enough, it is obviously better to accept that risk than to stick with high risk levels hoping that a way to achieve even lower risk will be discovered.

Harm reduction is particularly compelling for the use of nicotine because so many people have such a strong propensity for using it. Nicotine is a very beneficial drug for many people, providing alertness, focus, pleasure, and relief from a variety of psychological symptoms and pathologies. A substantial fraction of the population gets these benefits by smoking even though the health costs are so high, which means that demanding they quit entirely entails great welfare costs and is not likely to work.

Smoking can be described compellingly in terms of normal welfare economics, such that the consumer is maximizing his welfare by choosing among the available options (smoke or not smoke). Both choices have costs and benefits, and some consumers judge that the benefits of smoking outweigh its very high costs. However, for many such smokers, the possible reduction in benefits from switching to a less-enjoyed product would be greatly outweighed by the reduction in costs from health risks, so knowing about the benefits of switching to a THR product would be tremendously beneficial. Alternatively, it is often implicitly argued that smoking behavior does not conform to rational choice theory: Smokers do not choose smoking from among their options, but rather "addiction" (a rather slippery concept which is seldom actually defined, but is still widely invoked and accepted) or some related phenomenon prevents smokers from being able to choose to be abstinent. In that case, THR offers a health benefit that is not going to be achieved by choosing abstinence, and thereby also provides a great welfare benefit. Thus, either of these models of individual behavior leads to the same conclusion: Many people who are faced with the dichotomous choice of smoking and abstinence will not just quit, and many of them would be better off using nicotine in a low-risk form. Therefore, whether one believes that smokers are making a rational welfare-maximizing choice or are victims of a curse, THR makes sense from the perspective of both individual welfare and public health. (Further exploration of the policy-ethics arguments surrounding promotion of THR can be found in the collection of papers at .)

It might seem surprising that something as promising as THR is largely unknown and unimplemented as a policy. Much of the problem is that people (smokers, health educators, policy makers) hear the messages that THR products are not safe, that "all tobacco is deadly", and "the only safe choice is to quit entirely". This convinces people that THR either is not possible at all or represents only a marginal improvement that is not worth pursuing. Still, this begs the question of why anyone would choose to deliver the message that a 99% reduction in risk is almost as bad as continuing to smoke, rather than the obviously more accurate message that it is almost as good as quitting entirely. Answering this is useful for understanding the significance of the analysis presented here.

### Why analyses like this one are needed

The discourse surrounding tobacco policy and education is dominated by people who pursue the most extreme possible goal regarding tobacco: unconditional elimination of its use. Explicit statements of that goal are very common. Their goal is not to design tobacco policies that maximize human welfare or even that maximally reduce physical health costs. Any such concerns are, at best, secondary to the goal of simply reducing consumption of all forms of tobacco, and usually also reducing any long-term self-administration of nicotine that has been extracted from the tobacco (i.e., electronic cigarettes and pharmaceutical products). Thus, while getting smokers to switch to using ST represents an almost perfect success from the public health perspective (and is even more attractive from the human welfare perspective), it represents little or no progress for someone pursuing the goal of unconditionally eliminating tobacco use from the world. Presumably those who believe that eliminating tobacco is the appropriate goal would not dispute this. With this in mind, it is much easier to understand why some people reject a 99% reduction in risk as not worth pursuing: reducing risk is not the major factor in their objective function.

(This, of course, does not address the question of *why *anti-tobacco extremists are motivated to pursue this goal. Exploring possible explanations is beyond present scope (they are discussed in a bit more depth in Phillips, Heavner & Bergen (Phillips CV, Heavner K, Bergen P. Tobacco - the greatest untapped potential for harm reduction. Submitted, Available at: )). The list includes: the economically absurd belief that nicotine products provide no benefits and thus no one really wants to use them, usually closely tied to the paternalistic notion that the activists are better able to determine what people really want than the consumers themselves; an irrational hatred of companies who make nicotine products (often with the exception of pharmaceutical companies who many anti-tobacco activists are closely allied with); the common drug-war mentality of wanting to purify everyone and considering users to be sinners; and simple involvement of individual ego, whereby the goals becomes about winning the race and defeating the opponent, without ever admitting that their strategy may not have been optimal, rather than trying to develop humane, rational, practical policies.)

Understanding this is critical because those pursuing the extreme anti-tobacco agenda are often thought to have risk reduction as their primary objective, and take advantage of this by making dozens of health risk claims. It is, of course, people's right to hold the political opinion that we should work toward eliminating all tobacco use, regardless of how pursuing that goal would affect people's welfare and health, and it is those advocates' right to campaign for their goal. The ethical problems and public confusion result when the primary goal is eliminating tobacco, but the rhetoric mostly consists of claims about health. When such a disconnect occurs, the claims are merely rationalizations or attempts to persuade those who might not be persuaded by the true goal, rather than representing true underlying motives. When the language of science is used to rationalize rather than analyze, the probability is high that the science will degenerate into pseudo-scientific rhetoric.

None of this should come as a great surprise given the history of other abstinence-only agendas presented in the guise of public health. It has long been accepted by the public health community that harm reduction strategies for illicit drug use, from needle exchanges to education about the advantages of moderation, save many lives. Nevertheless, anti-drug warriors who support a "just say no"-only strategy frequently try to shut down programs that promote harm reduction. Their explicit argument is never "those criminals deserve to die if they do not quit using drugs, so we should not try to lower their risk"; in fact, their public argument is often based on inaccurate claims that the harm reduction strategies increase risk. Similarly, it has been known for decades that abstinence-only approaches to sex education in the West produce inferior health outcomes compared to balanced harm-reduction-oriented education, combined with product and service provision. Activists who persist in claiming that promoting only sexual abstinence is health-improving seem to not be concerned with health so much as they are just annoyed that people are enjoying sex outside of marriage.

The politics and rhetoric of the abstinence-only approach to nicotine use have much in common with these other abstinence-only approaches, but this is not yet widely recognized. As a result, many people who are genuinely motivated by promoting personal and public health, and do not share the extreme anti-tobacco agenda, often believe the inaccurate health claims that are really rationalizations for the anti-tobacco position. Since this often is to the detriment of both public health and the scientific legitimacy of the health sciences, it is important for the public health and scientific communities to debunk these claims.

Debunking these claims is a difficult challenge. Anti-THR health claims are typically speculation or assertion, without the support of evidence or analysis, and thus actual scientists will immediately relegate them to the realm of, at best, speculative hypothesis. But it is easy to take advantage of laypeople's tendencies to accept at face value all manner of urban myths and other misconceptions, and to demand scientific proof that the claim is wrong [11]. Endeavoring to disprove a long list of assertions is far more difficult than making up those claims in the first place. Indeed, the sheer number and ever-changing nature of those claims is further evidence of attempts to rationalize a pre-determined conclusion, not an exploration of real reasons: Generally when someone shops different claims to various populations to see which changes their behavior in the preferred way, we call it marketing, not science, education, or ethical public health policy.

### Methods of responding to misleading claims

But though trying to disprove unsubstantiated claims is not considered necessary in scientific thinking and is obviously an epistemic nightmare, it is necessary to advance public health policy. Advocates of THR have endeavored to debunk some of the most erroneous anti-THR claims. Some claims have been debunked by simply pointing to existing scientific literature (e.g., claims that ST use causes substantial disease risk are contradicted by decades of epidemiologic evidence to the contrary). Some claims have required new directed empirical work (e.g., the claim that promoting THR would create a "gateway" to smoking required focused empirical research and analysis to debunk). Still others are hypothetical scenarios that require an analytic approach to show they are misleading or of minor consequence.

An example of such analysis is the debunking of the claim that if we allow smokers to learn that they have low-risk alternative sources of nicotine, then many people who might have had zero risk from consuming nicotine (because they would have quit entirely or not started) will choose to consume ST or pharmaceutical nicotine and suffer some small risk. This will, the claim goes, increase total population risk. But when it is demonstrated that net social risk could not conceivably increase in this manner, anti-THR activists sometimes counter with a second assertion: Even though total population risk will decrease, there are many smokers who would have quit nicotine entirely but instead switch to a low-risk product, and they will suffer greater risks than they otherwise would, and that this constitutes an argument against THR. Debunking this requires the additional analysis presented below.

One might argue that the ethical considerations make quantifying this claim irrelevant. The leading deontological tenet of modern health ethics is the obligation to provide people with accurate information so they can make informed autonomous decisions about their own health. Thus, whatever one might think about actively promoting THR as public policy, it is *per se *unethical to mislead people in order to manipulate their health behavior, even if it is "for their own good" (Phillips CV. The affirmative ethical arguments for promoting a policy of tobacco harm reduction. Submitted, Available at: ). In other words, preventing a smoker from learning about a low-risk alternative, even if he is about to quit entirely, is clearly unethical. Moreover, a consequentialist analysis reveals that someone who chooses to forgo nicotine because of the high cost of smoking but, upon learning of a low-risk way to consume nicotine, chooses to consume low-risk nicotine must have concluded that the net welfare benefits of consumption (the benefits of nicotine, net of the health and other costs) are positive, even though the net benefits of smoking were negative. Therefore misleading people about the option necessarily has net negative welfare impact (Phillips CV. The affirmative ethical arguments for promoting a policy of tobacco harm reduction. Submitted, Available at: ).

Nevertheless, some observers are unconcerned with these ethical arguments. More importantly, the claim brings up an interesting analytic question that is worth answering even apart from the politics of THR: In terms of physical health risks, someone who keeps smoking is clearly worse off than someone who switches immediately, who in turn is probably slightly worse off than someone who immediately quits entirely. But how long would someone have to keep smoking before his health risks would have been lower had he just switched today and used low-risk nicotine for the rest of his life? Or, equivalently, how much time can pass while powerful interests vilify THR products while waiting for theoretical perfect alternatives to emerge before that delay kills as many people as using THR products ever could? For anyone who is primarily concerned about maximizing health outcomes (even apart from rights to autonomy or welfare maximization), the answer to these questions should make it clear that THR should immediately be embraced using currently available alternative products.

## Analysis

It is illustrative to begin this analysis by addressing the assertion that total social (population) risk will increase if THR is embraced, explaining how that is insupportable, before continuing to the new analysis of the individual smoker who will either switch or quit.

### Net effect on social risk of lowering individual risk

It is clear that lowering the risk from consuming nicotine (or, more precisely, making people aware of the fact that they have the option of lowering their own risk) should result in some people using nicotine who otherwise would not. Simple economics tells us that when the population learns that they can receive the benefits of nicotine with much lower total cost (due to almost eliminating the health risk), rational behavior causes increased consumption. This means that demands like the Society for Research on Nicotine and Tobacco's (SRNT) policy statement, " [THR] should not reduce the likelihood of eventual cessation of tobacco use" and "should not lead to increased population prevalence of tobacco [use]" [12] are tantamount to saying that any step that lowers the risk from using tobacco - whether it be creating a safer product or finding a cure for lung cancer - is unacceptable. This is critical to understand: Finding a cure for lung cancer would inevitably increase the number of people who smoke, and thus the SRNT is demanding that no such cure be pursued. More generally, insisting that a health policy or technology, even one that saves many lives, is only acceptable if it does not lead to an increase in the number of people engaging in risky activities would not only forbid THR, but would also prohibit condoms, sports safety equipment, sunscreen, lifeguards, vaccines for travelers, and trauma centers.

In fairness, those who make such statements are probably not intentionally calling for a prohibition against lowering the risks from smoking, such as by demanding that we avoid curing cancer. They are probably just ignorant of basic economics and how changing costs influence people's decisions. Though there are skilled economists involved in "tobacco control" research and advocacy, they seem to have done little to educate or influence activism or policy statements. The most vocal activists are clearly unaware of the overwhelming economic evidence about how individuals optimize consumption, or reject that evidence without any basis for doing so, and thereby reject the liberal ethics of economics-based consumer policy that follow from it. This is not merely a matter of considering individual smokers as irrational, since it even extends to assuming profit maximizing businesses do not follow their best interests - e.g., they insist that prohibiting a popular voluntary commercial choice, banning smoking areas in pubs, does not merely result in a net health improvement, but actually never hurts any merchant [13]. However, even though economic ignorance is a compelling explanation, we cannot rule out the possibility that many anti-tobacco extremists really mean what they say, and actually favor maximizing the risk from using nicotine and otherwise intentionally lowering people's welfare in order to make tobacco/nicotine use less appealing.

Empirical support for the economic prediction that lowering risk will increase consumption (either by more people consuming the good, or those who are consuming it using more, or both) can be found in Sweden. Most Swedish would-be-smokers (particularly men, but increasingly women also) use ST instead, resulting in by far the lowest consumption of smoked tobacco in the Western world. The result is the expected reduction in smoking-caused diseases, with no offsetting increase in ST-caused diseases (which is to be expected, since no detectable level of any disease has been shown to be caused by ST). But total tobacco consumption in Sweden is among the highest in Europe. Anti-tobacco extremists, therefore, consider the Swedish experience to represent a failure, consistent with their political goal of reducing tobacco use regardless of the health effects. Realizing, however, that most observers would not share that goal, they try to rationalize their position that this public health triumph is really a failure by trying to deny the public health gains.

Indeed, it should be recognized as a reassuring observation about people to see that when the health risk from a consumption choice is dramatically reduced, people rationally increase total consumption. Many readers will probably find it odd to declare it reassuring that more people would become nicotine users, but a single observation should be sufficient to eliminate all confusion: The prediction that some people who would not smoke will choose to use low-risk nicotine products is equivalent to the more politically correct statement, "some people choose to avoid smoking due to the high health costs even though they would like to get the nicotine." Few would disagree that the latter is a reassuring observation about people's rationality.

Extending this, it is plausible that lowering the health risks of consuming something could increase consumption to the point that the total social risk will increase. It must be the case that there is an improvement in total net social benefits, since the change would result from free choice of a preferred option, and the major externalities would likely also be positive. But health risk, considered apart from other contributors to welfare, might increase. All that is necessary for an increase in health risk is that the quantity consumed goes up by enough that even with the lower risk, the total risk (i.e., quantity consumed multiplied by average individual risk per unit of consumption or, in units of people, the number of consumers multiplied by the average risk per consumer) is greater. Whether this happens in a given case is an empirical point, but for the case of smokers and some nonsmokers adopting a low-risk nicotine product, a simple analytic reality check shows that it is effectively impossible.

Given the estimate that switching to a low-risk alternative reduces a smoker's risk by 99%, if only 1% of a population switched from being continuing smokers to using THR products, then even if the entire rest of the population switched from no consumption to the low-risk products it would not result in a social risk increase. (The number of additional users necessary to make up for the risk decrease from one switcher is easily calculated as (1-x)/x, where x = the proportion of the risk from smoking caused by the THR product, so since (1-.01)/.01 = 99, then for 1 smoker who switched from smoking, there would have to be 99 non-users who took up ST to make up for it.) Even if the alternative product was 5% as harmful as continuing to smoke, which is difficult to imagine given the available evidence, if 1% of the population switched (which would represent less than 5% of all smokers in Western populations, a very modest success), the new product would have to attract 19% of the population, roughly one-quarter of all current non-users, to start using nicotine in the low-risk form to result in no net gain. This would represent total nicotine usage prevalence close to the maximum it ever reaches, even in populations not worried about health risks, which is presumably the total portion of the population that benefits from using nicotine. Thus, even a pessimistic comparative risk scenario leaves little room for an increase in social health risk.

The argument that total population risk might increase and therefore we should not inform people about THR - though arithmetically absurd and based on the unethical premise that it is acceptable to mislead people - has proven to be a remarkably persistent rationalization for anti-THR activists. It is so often repeated that the original debunking of it, an article that basically just graphs the y = (1-x)/x function and expands on the point from the previous paragraph [14], has been cited by scores of journal articles about THR (including most of the substantive overview articles on the topic) and hundreds of presentations and popular communications, presumably because the later authors believed it was necessary to respond to the claim that the article debunks. But there has not previously been a good quantitative response to the next layer of rationalization: Even though social risk will clearly be lower if THR is widely adopted, somewhere out there is a hapless smoker who would have soon won his struggle to give up nicotine to avoid all further health cost, but he becomes doomed to failure when presented with the information that he could use a low-risk alternative, resulting in a net health cost.

This claim, plausible until one actually checks the numbers, typically takes a form like THR "may undermine efforts leading to the healthiest outcome of all, namely, complete tobacco abstinence". Versions of this claim are common in statements made to the popular press by anti-THR activists and in rhetorical documents put out by anti-tobacco extremist organizations (though this particular quotation actually comes from an ostensibly scientific journal article [12]). Setting aside the inappropriate breadth of this phrasing (it is generally accepted that "healthiest" should incorporate psychological health, not just longevity, and since nicotine has substantial psychological benefits, abstinence is often not healthiest), the implicit claim is quantitative and a function of the time periods involved. Claiming that the outcome the authors personally prefer, abstinence, is healthiest (in the narrow sense of maximizing life expectancy) depends on the implicit quantitative claim that the hypothetical complete cessation of nicotine use would have begun soon enough that it would have resulted in less physical health risk than consuming a low-risk alternative. (Some might claim that such authors are merely suggesting that immediate abstinence would be the physically healthiest behavior, without reference to what might actually happen. But this defense is not convincing since the statements are made in the context of policy recommendations and other practical discussions, where obviously no one would suggest that assessing the effect of universal immediate abstinence has any practical relevance. After all, if the authors merely wanted to make a statement about what would be best, without regard to what is actually possible, then making it so that no one ever smoked in the first place would actually be best.)

Sometimes the claim is made in a form that practically concedes that eliminating tobacco use (and often any close substitute for it, like electronic cigarettes), rather than improving health, is the author's primary goal (e.g., "The major concerns of promoting a dangerous product as less harmful than another are that it may undermine efforts to achieve total tobacco-product cessation" [15]). However, such claims are typically presented in a way to imply that readers concerned with health outcomes should consider them to be health-based (in the previous example, the assertion appeared under the heading, "public health implications of the findings from this study"). But even authors editorializing a pro-THR position, and thus presumably not basing their views on the anti-tobacco extremist position, often suggest that a "downside" [16] of having the option to switch will cause some people who would have quit entirely to suffer greater risk because they switch instead. But how many potential quitters actually fall into this "downside"? That is, how many were going to quit soon enough that switching actually represents a net increase in disease risk?

### Calculation of the switch-versus-eventually-quit tradeoff

The following analysis quantifies the question about "soon enough". Note that this calculation addresses only the risk-risk tradeoff, ignoring any benefits of continuing to use nicotine rather than quitting and the welfare costs of the act of quitting. It is also limited to mortality even though non-fatal morbidity is probably not perfectly proportional to mortality risk. The latter simplification, as well as the necessarily rough input numbers, are relatively minor compared to the simplifications that exist (though are seldom acknowledged) in most population health analyses. More important, they prove to matter little, given the clear implications of the result. This analysis proves to be an excellent example of the value of a back-of-the-envelope calculation as adequate response to an unanalyzed claim: While it is often not practical to complete a precise analysis of a scientific or policy claim, it is often the case that the rough analysis that is practical is quite adequate for present needs, and is a great improvement over unquantified speculation.

For any given smoker at a particular time, who is not already doomed to die from his smoking to date, we wish to estimate how many days of continuing smoking causes as much risk of death as a future lifetime of using a low risk nicotine product. (Note: describing something as causing someone's death is shorthand for saying that it substantially hastened the death, and obviously not that ever-dying was conditional on the behavior.)

Answering the question for an individual would require determining the probability of dying from a lifetime of THR product use, starting at the present, and the probability of dying from future smoking as a function of how long the smoking continues. While it would be useful to have such a lifecycle-based model for individual decisions, it is not currently possible. An individual's risk from a lifetime of THR product use could be reasonably estimated as a function of the individual's current life expectancy, with possible refinement by inclusion of other variables. But despite the extensive research on smoking and health, there is apparently no good calculation of the risk from a short future period of smoking, based on current age, sex, etc. There is ample research about the benefits of quitting and it clearly establishes that quitting sooner is better, but it offers very limited information for calculating the marginal cost of a given additional period of smoking as a function of past smoking duration and other individual characteristics. Thus, while comparative observations are possible based on the demographics of the individual in question (e.g., a very young smoker, with a long potential period of THR product use, has more to lose from switching rather than quitting after a particular delay, and thus could afford a longer wait until quitting), there is currently no realistic way to do this calculation for individuals.

But from the public health education and policy perspective, knowing the risk-risk tradeoff on a population average basis is almost as useful, and calculating that is possible. The population average can be viewed as comparing switching-now-versus-quitting-later for all smokers acting simultaneously (which, of course, will not happen - it is just a useful unit of analysis) or, equivalently, asking the question for a random smoker we know nothing about. Public health interventions, particularly the provision of information, typically affect all or random individuals, making this the relevant level of analysis.

The key to the calculation is the observation that if we assume that smoking more never cures a disease that was caused by previous smoking, then for anyone who dies from smoking, there will be a day, D, in his smoking history such that if he had quit entirely before that day he would not have died from smoking, but as a result of smoking through that day he does die from smoking. Because we never know which day that is, and because smoking-caused disease results from an accumulation of insults, this observation may not be obvious to all readers. For those who do not find this observation intuitive, a simple proof follows.

*Proof*: Assume that a destined-to-be-fatal disease that was caused by past smoking is never cured or delayed by future smoking. Consider someone who dies from smoking. Consider the latest day, if it exists, of smoking during his life such that had he quit entirely before that day he would not have died from smoking. Since this is the latest such day and he did die from smoking, if he smoked that day he would still have died from smoking, which defines day D. The smoker's life was finite, and thus includes a finite t days of smoking. Had he quit just before day t, either he would have still died from smoking (either from the disease that actually killed him or another disease also caused by smoking) or not. If not then day t meets the definition of D (if he had quit the day before he would not have died, and t is necessarily the latest such day). If day t is not D, then either he would have not died from smoking if he he had only smoked through day t-2, in which case day t-1 is D (if he had quit before that day he would not have died, and this is not true for any later day). If t-1 is not D then a similar analysis can be applied to t-2, and so on. Thus, by counting down through the finite list of days, we either find some day that is D or reach day 1 without having found D, in which case quitting any time after day 1 would not have stopped the death from smoking. But by hypothesis the death was caused by smoking, so never starting (quitting before day 1) would have prevented it, and therefore day 1 is D. Therefore, D exists sometime within the days of smoking for each individual who dies (or is destined to die) from smoking.

The same logic proves that for every smoker who dies of smoking there was one particular cigarette that was the fatal point-of-no-return. The proof does not address the fact that moving toward quitting might alter which day is D by altering smoking intensity or starting and stopping. It also ignores the possibility that further smoking past D could further accelerate the death from smoking, making the subsequent analysis conservative because it ignores the possible longevity benefits of switching among those already doomed to die from their smoking.

Given that everyone who dies from smoking has a D, it is possible to estimate the increased risk of dying from smoking for the average smoker (or all smokers) from smoking one more day. For a typical Western population, we can estimate the average lifetime days of smoking for someone who dies from smoking to be about 18,000 (about 50 years). Since one of those days must be D, the average day of smoking from someone who is destined to die from smoking (averaged across all days of smoking among all such individuals) has probability 1/18,000 of being the day that doomed the smoker to die from smoking. Thus, if all current smokers who are destined to die from smoking gave up smoking tonight, some number, x, of them would be saved from dying from smoking, but if instead they gave up smoking tomorrow night, only x minus 1/18,000th of that population would be saved.

Notice one immediate observation based on this that is apparently not obvious to many smokers and people who give advice on these matters: Quitting *someday *is not sufficient - it is possible to quit too late and there is no way to know in advance which day is one day too late.

Estimates for Western populations of the fraction of current smokers whose deaths will be caused by smoking range from 1/4 to 1/2, so roughly one death from smoking is caused by each 50,000 days of smoking. The best available estimate is that the average risk of dying from THR product use is about 1% that from smoking. Following the above logic, this represents 5×10^6 ^days of use per death caused. Since the ratio of the risk from THR product use compared to smoking enters the calculation linearly, readers who believe the ratio is really 2% or 3% can adjust the final estimates upward by a factor of 2 or 3. (Readers who believe the ratio is much more than that should take a closer look at the scientific evidence.) Assume that the total risk from THR product use is the same whether it is a lifetime of exclusive THR product use or switching to THR products after some period of smoking. Note that this is a conservative assumption, since any smoker who is already doomed to die from smoking experiences no increase in the chance of dying from nicotine use by using a THR product. Moreover, it seems fairly likely that if THR product use causes any negative health impacts other than the minor effects of nicotine itself, then they are not exactly the same as those from smoking, and so the additive health effect of THR product use on top of smoking would probably be less than the additive effect of a longer term of THR product use.

We can estimate that if smokers who are going to eventually cause themselves to die from smoking will smoke an average of 18,000 days, then the average such current smoker has about 9,000 days of smoking ahead of him. (This is would be exactly true if we were in steady-state with respect to smoking and if smokers with fewer days of smoking ahead of them were not more likely to already be doomed. Failures of these assumptions will tend toward canceling out, and the net error seems to be within the limited precision built into the calculation.) Thus, using the conservative simplification above, if the average such smoker switches immediately, he has a 9,000/5×10^6 ^≈ 1/600 chance of dying from ST use. Comparing this to his extra probability of dying from smoking by waiting longer to completely quit, at 1/18,000 chance of causing death per day, shows that this is the equivalent of delaying quitting by about one month. Thus, on average, this smoker only endures greater total risk from using a THR product for the rest of his life if he were going to become abstinent in less than a month.

Note that the "all smokers" or "randomly selected individual" condition is crucial here since, for example, a particular smoker who is young and therefore has not yet smoked much can probably get away with smoking years more before being doomed, but has many more days of potential THR product use ahead of him, might not reach risk parity for several months. Conversely, there are older demographic groups, possibly identifiable, who may not yet be doomed but are much more likely than average to be close, for whom a single additional day of smoking poses greater risk than a future lifetime of THR product use.

## Discussion

While it is logically possible that lowering the risk from an exposure could increase population risk, the (1-x)/x calculation shows this is not plausible for THR. The suggestion that, despite the lower population risk, many individuals might still face greater risk is also logically possible, but the calculation presented here shows that this is not a substantial practical worry.

On average, someone who would die from smoking who is going to take more than a month to quit entirely (or will experience relapses that will have a similar health impact - probably roughly a total of one month worth of days) will have less total health risk by switching immediately, even if he never quits the alternative product. The typical pattern of even dedicated quitters, starting and stopping smoking for a year or two, will cause much more risk than switching to a low-risk alternative. Moreover, even an average smoker who was going to successfully quit after only a week or two more will suffer only a tiny net increase in physical health risk from switching now, a change so trivial compared to the net benefits of switching for smokers who will not quit for years or ever that it is clearly inconsequential.

The practical implications of this analysis do not change based on plausible variations in the input parameters, including the risk from using ST. Even if we use a completely implausible high risk from ST use, say that it causes 10% of the risk of smoking, then if an average smoker would have taken ten months to quit entirely, he would have had lower risk had he switched immediately. The break-even might be as low as about half a year - recall the conservative assumption built into the calculation. Thus, even discovering that ST use is an order of magnitude worse than the ample current evidence suggests would not fundamentally change the implications of the analysis.

Since this analysis is based entirely on mortality risk, it ignores other contributions to welfare. The reason that current smokers have not already quit, in spite of the health benefits of doing so, is that it would have resulted in substantial costs to them and, similarly, whenever a smoker chooses to switch it implies that there is a net welfare benefit (compared to either smoking or abstinence) to using the alternative product. This welfare gain from switching rather than quitting probably dwarfs the welfare implications of the mortality risk from low-risk products, though quantifying that is beyond the present scope.

Finally, it is worth noting that someone who switches from smoking to a low-risk alternative still has the option of quitting entirely, lowering his risk slightly more still. Indeed, there is reason to believe that eventually quitting alternative products is easier. This means that even the young smokers who might have been better off with several more months of smoking rather than a lifetime of THR product use stand a good chance of quitting entirely anyway (if they decide that the benefits of consumption are outweighed by the benefits of quitting), further favoring the option of switching now. Even those smokers who cannot afford another day of smoking but fortunately switch just in time (who are likely from older demographics that are the primary target for THR) could then survive long enough to quit nicotine entirely.

Many of the claims about health risk made to try to discourage the adoption of THR have been proven to be out-and-out false. This includes the "total social health risk will increase" claim. The present analysis does not relegate the "some people would be stopped from quitting entirely and thus have worse health outcomes" claim to universal falsehood - it will still inevitably be true for a very few individuals. But this is common in public health interventions, from automobile safety equipment to vaccines - the net social effects are overwhelmingly beneficial, though some people (who cannot be identified *ex ante*, and often not even *ex post*) suffer net harm rather than benefit. The analysis shows that only a tiny portion of all future quitters will be quitting soon enough that they would have higher expected risk by switching immediately. Moreover, the net increase in expected risk even for those individuals would be extremely small, and the net welfare effects would still be positive. Clearly, then, the claim does not represent a sufficient concern to override the huge net expected social benefit, to say nothing of the ethical requirement that smokers be informed about their options. The claim is thus relegated to being a distraction from rational and honest discourse on the subject, not a contribution to it.

This calculation emphasizes the cost of delaying the adoption of THR at the individual level also: Those of us who promote THR are familiar with smokers who, upon learning about THR, insist that they do not need to consider that option because they will eventually be exercising the "perfect" option of quitting anyway. But many such individuals never quit, and almost none quit in time for it to be a healthier choice. Similarly, each additional month that anti-THR activism keeps a potential switcher from learning about THR is more likely to kill him than is a lifetime of using ST or another low-risk nicotine product. To put it bluntly, anti-THR activism and disinformation do far more damage to public health than smokeless tobacco, electronic cigarettes, or other THR products ever could.

Since THR can be self-tailored and requires no clinical or government intervention, it does not matter that there may be smokers for whom no low risk product is an adequate substitute or that there is no political will to actively endorse it. THR can be adopted by individuals who do find an acceptable substitute, and likely will be widely adopted if smokers were simply given accurate information. The usual explanation for the lack of such information is that anti-tobacco extremists promulgate disinformation it and then even the opinion leaders who are genuinely concerned about public health repeat the inaccurate claims because they have been misled. But an alternative explanation is misplaced optimism on the part of the public health leaders: That is, many may not be misled by the disinformation about THR, but may genuinely believe that most smokers will successfully quit using nicotine very soon or that a perfect new anti-smoking method, policy, or product will be developed and cause everyone to quit soon, reducing their risks more than THR would. The present analysis shows just how overly-optimistic that belief needs to be in order to justify the failure to immediately promote THR using current technology.

Whatever the explanation for it, the present analysis shows that anti-THR activism is deadly. Hiding THR from smokers, waiting for them to decide to quit entirely or waiting for a new anti-smoking magic bullet, causes the deaths of more smokers every month than a lifetime using low-risk nicotine products ever could.

## Competing interests

The author is an advocate of tobacco harm reduction, and thus has worldly goals that are furthered by debunking anti-THR rationalizations. He is also interested in improving research in public health and promoting evidence-based public policy, and thus has an interest in calling attention to flawed reasoning. In particular, he has long taught his students the value of back-of-the-envelope analysis and related reasoning, and so is motivated to seek examples that demonstrate its usefulness. The author has been the target of a well-documented campaign of attacks by anti-THR activists trying to damage his career and force him to stop doing THR research [17]. While nothing in this paper is a specific response to those attacks (the worst attacks have come mostly from minor local activists and the administration of the University of Alberta School of Public Health, not the internationally-known political activists cited in this paper), anyone who takes the concept of competing interests seriously will realize that such personal experiences may motivate behavior in ways an individual is not consciously aware of. The author's research is partially supported by an unrestricted (completely hands-off) grant to the University of Alberta School of Public Health from U.S. Smokeless Tobacco Company; the funders have had no input into the design or content of this analysis, and were not aware of it until it was made available to the public. Far more importantly, this author, like almost all other health researchers, is dependent on getting future funding from someone, future positive peer reviews, etc., if he is to continue his research, which in this case creates the conflicting incentive to push the frontiers in supporting the wisdom of THR (to make the research agenda more accepted) and for minimizing confrontations with powerful interest groups (to make himself more acceptable). The author advises many organizations on tobacco harm reduction, some of which are companies that profit from selling nicotine products, and is sometimes compensated for his time. In addition, he consults for USSTC in the context of litigation, has minor financial interests in the financial health of certain nicotine product manufacturers, occasionally uses several of the products mentioned in this paper, and has friends who have no intention of ever quitting their use of nicotine.
